# An Expert-Based Review on the Relevance and Management of Type 2 Endoleaks Following Endovascular Repair of Ruptured Abdominal Aortic Aneurysms

**DOI:** 10.3390/jcm13154300

**Published:** 2024-07-23

**Authors:** Philip Dueppers, Mario D’Oria, Sandro Lepidi, Cristiano Calvagna, Alexander Zimmermann, Reinhard Kopp

**Affiliations:** 1 Department of Vascular Surgery, University of Zurich (UZH), Raemistrasse 100, CH-8008 Zurich, Switzerland; alexander.zimmermann@usz.ch (A.Z.); rk_kopp@web.de (R.K.); 2 Department of Vascular Surgery, Kantonsspital St. Gallen, CH-9000 St. Gallen, Switzerland; 3 Division of Vascular and Endovascular Surgery, Department of Clinical Surgical and Health Sciences, University of Trieste, 34149 Trieste, Italy; mario.doria88@outlook.com (M.D.); slepidi@units.it (S.L.); cristianomaria.calvagna@asugi.sanita.fvg.it (C.C.); 4 Department of Vascular and Endovascular Surgery, University Hospital Regensburg, University of Regensburg, 93053 Regensburg, Germany

**Keywords:** abdominal aorta, endovascular aortic repair, ruptured abdominal aortic aneurysm, lumbar arteries, type 2 endoleak, therapeutic embolization, coiling

## Abstract

Ruptured abdominal aortic aneurysms (rAAAs) are life-threatening and require emergent surgical therapy. Endovascular aortic repair for rupture (rEVAR) has become the leading strategy due to its minimal invasive approach with expected lower morbidity and mortality, especially in patients presenting with hemodynamic instability and relevant comorbidities. Following rEVAR, intraoperative angiography or early postinterventional computed tomography angiography have to exclude early type 1 or 3 endoleaks requiring immediate reintervention. Persistent type 2 endoleaks (T2ELs) after rEVAR, in contrast to elective cases, can cause possibly lethal situations due to continuing extravascular blood loss through the remaining aortic aneurysm rupture site. Therefore, early identification of relevant persistent T2ELs associated with continuous bleeding and hemodynamic instability and immediate management is mandatory in the acute postoperative setting following rEVAR. Different techniques and concepts for the occlusion of T2ELs after rEVAR are available, and most of them are also used for relevant T2ELs after elective EVAR. In addition to various interventional embolization procedures for persistent T2ELs, some patients require open surgical occlusion of T2EL-feeding arteries, abdominal compartment decompression or direct surgical patch occlusion of the aneurysm rupture site after rEVAR. So far, in the acute situation of rAAAs, indications for preemptive or intraoperative T2EL embolization during rEVAR have not been established. In the long term, persistent T2ELs after rEVAR can lead to continuous aneurysm expansion with the possible development of secondary proximal type I endoleaks and an increased risk of re-rupture requiring regular follow-up and early consideration for reintervention. To date, only very few studies have investigated T2ELs after rEVAR or compared outcomes with those from elective EVAR regarding the special aspects of persisting T2ELs. This narrative review is intended to present the current knowledge on the incidence, natural history, relevance and strategies for T2EL management after rEVAR.

## 1. Introduction

Abdominal aortic aneurysms (AAAs) have a prevalence of up to 8% in men above the age of 65 years [1]. It is a leading cause of mortality if rupture is not prevented by surgical treatment at a threshold diameter of 55 mm in male or 50 mm in female patients [1]. Current guidelines recommend elective endovascular aortic repair (eEVAR) as the first-line option for most patients with suitable anatomy and reasonable life expectancy due to its lower morbidity and invasiveness over open surgical repair (OSR). The same recommendation applies for ruptured endovascular aortic repair (rEVAR) in patients with a ruptured AAA (rAAA) based on large randomized controlled trials [2,3,4]. These trials suggested several advantages of eEVAR over OSR including lower mortality, faster discharge, a gain in quality-adjusted life years and reduced costs. As a consequence, eEVAR is used in up to 80% and rEVAR in up to 60% of cases [5]. To achieve a rapid hemodynamic control, emergent aortic balloon occlusion before either surgical strategy is recommended [6]. Nonetheless, OSR for rAAAs is still a valid option if morphology does not allow an endovascular procedure [7].

However, long-term outcomes of endovascular repair are characterized by a higher need for reintervention in comparison to OSR [8]. The majority of these post-EVAR reinterventions are related to the occurrence of endoleaks, which are classified according to their origin [9]. A type 2 endoleak (T2EL) occurs when blood flows in a retrograde direction into the aneurysm sac. T2ELs are further divided based on the source of blood flow into type 2A (from lumbar arteries), type 2B (from the inferior mesenteric artery (IMA)) and type 2C (from other sources). After eEVAR, the occurrence of T2ELs is described in a wide range between 6 and 44% of cases [10,11,12,13]. They are generally considered less immediate threats than their type 1 (T1EL; leakage at the proximal or distal sealing zone) and type 3 (T3EL; leakage at the connecting sites of stent–graft components or through discontinuity in the stent–graft fabric) counterparts and more than 30% resolve spontaneously while an even larger proportion will not lead to any sac increase over time despite the absence of spontaneous resolution [14]. If T2ELs persist and lead to an aneurysm sac enlargement of >1 cm within 12 months as compared with the pre-intervention baseline measurements, surgical treatment is recommended. This primarily involves endovascular means using various embolization techniques to prevent subsequent complications such as sac expansion and/or secondary rupture. If the aneurysm sac diameter does not increase, frequent follow-up using contrast-enhanced ultrasound (CEUS) or computed tomography angiography (CTA) is indicated [15,16].

While these recommendations are well established for T2ELs after eEVAR, data on T2ELs after rEVAR are limited and only a few relevant studies and case reports have been published [12,17,18,19,20,21]. In effect, it may be counterintuitive not to treat a continuous (although low) sac flow in the setting of rAAAs, as this may lead to ongoing blood loss and contribute to hemodynamic instability. However, given the immediate life-threatening nature of rAAAs, it should be borne in mind that a “damage control-like” approach is to be endorsed, which entails the immediate endovascular or surgical treatment of the aortic rupture to stop continuous blood loss through the rupture site. Therefore, the additional time needed to try and achieve the simultaneous or early treatment of T2EL after rEVAR must be balanced against more pressing concerns to the early restoration of patients’ hemodynamic stability.

This expert-based narrative review presents in a comprehensive way the current knowledge on the incidence, natural history, relevance and management of T2ELs after rEVAR.

## 2. Incidence and Diagnostics of T2ELs after rEVAR

The incidence of T2ELs after rEVAR is reported in up to 9–29% of patients [12,17,18]. Most T2ELs after rEVAR are supplied by iliolumbar vessels (75%), followed by the IMA (19%) or both sources (6%) [22].

Notably, a direct comparison between rEVAR and eEVAR by Quinn et al. revealed a significantly lower incidence of T2ELs after rEVAR (9% vs. 20%) [12]. This is interesting because one would assume that the rate of T2ELs after rEVAR would be higher due to an observed coagulopathy and hyperfibrinolysis in patients with an rAAA [23]. The author considered the hemodynamic collapse secondary to aneurysm rupture and blood loss as well as the usually waived perioperative anticoagulation with spontaneous thrombosis of lumbar arteries as possible reasons for the lower incidence of T2Els after rEVAR [12]. Other potential factors include stress-induced vasoconstriction and shock as well as endogenous und exogenous catecholamines.

One of the reasons why other studies have observed lower incidences of T2ELs after rEVAR as compared to eEVAR may be the challenge of postoperative T2EL visualization. Boniakowski et al. reported that 25% of T2ELs after rEVAR were not visualized on the completion angiograms and finally diagnosed only by postoperative CTA, which highlights the importance of a direct postoperative CTA after rEVAR [22]. Additionally, limiting factors associated with the non-optimal quality of fluoroscopic imaging like obesity, the presence of significant bowel air and, especially in rAAAs, large retroperitoneal hematoma can influence the detection rate, particularly of small T2ELs. Therefore, a second plane for the better visualization of persistent endoleaks during the final angiogram after rEVAR is usually recommended, considering the serum parameters for critical kidney function.

Follow-up imaging is usually conducted with CTA for objective but static images and/or contrast-enhanced ultrasound as a dynamic, but highly investigator-depending examination. The advantages of both modalities (objective and dynamic images) are combined in 4D MRI exams or dynamic perfusion CT scans as two recent advantages for the detection of even low-flow type II endoleaks [24,25]. 

Both have shown promising results, but so far have neither been examined in larger studies nor recommended in the current guidelines.

## 3. Risk Factors

The rEVAR-focused study by Boniakowski et al. described an association between the development of T2ELs and the body mass index. Interestingly, warfarin use, aortic thrombus burden and device type were not associated with T2EL development in that study [18].

In eEVAR patients, the presence of a metabolic syndrome, which usually corresponds with an elevated body mass index, and anticoagulation in combination with aspirin, which is usually prescribed post-EVAR, have also been associated with the development of T2ELs. The authors suggested the proinflammatory and prothrombotic state with associated endothelial dysfunction and other systemic changes in patients with metabolic syndrome as an explanation for this association [26,27].

Many other risk factors for the development or persistence of T2ELs after eEVAR are reported such as the total number of patent lumbar arteries, an aortic sac thrombus burden of <50%, hypogastric artery coil embolization, distal graft extension and age ≥ 80 years, graft type and absence of COPD [28,29,30]. Other studies described an association with the diameter of the IMA with the occurrence of T2ELs with a higher risk above a diameter of 2.5 mm [31,32], although there seems to be no clinical benefit after routine IMA coil embolization before eEVAR in terms of T2EL-related reintervention rates [33].

In conclusion, there is not enough evidence for any of these factors to recommend preemptive T2EL embolization either prior to eEVAR or to rEVAR.

## 4. Natural History

T2ELs after rEVAR possess a benign character in most patients [17]. As in eEVAR, they show a high rate of spontaneous resolution over time with reported rates of spontaneous closure up to 40–43% after around 3.5 months [17,18]. This sealing is associated with a significantly higher rate of aneurysm shrinkage of more than 5 mm compared to patients in whom the T2ELs persist [17].

In contrast to eEVAR, the aneurysm sac after rEVAR remains with its rupture site—if it was not secondarily repaired during decompressive laparotomy for abdominal compartment syndrome (ACS). This is the underlying reason why T2ELs after rEVAR—despite their usually benign course—can lead to even lethal persistent or new-onset hemorrhage in the early postoperative phase [17,18,19,21].

Another very peculiar, albeit fortunately rare, situation may be the presence of an aorto-caval fistula as an aneurysm rupture site with concomitant persistent T2EL after rEVAR [34,35]. In these patients, the T2ELs originate from the inferior mesenteric artery (IMA) with continuous retrograde high-pressure blood flow into the inferior vena cava frequently leading to progressive right cardiac decompensation or impending left colon malperfusion caused by the retrograde IMA shunt. In these patients, the hemoglobin levels remain normal although the patient becomes progressively hemodynamically instable. Endovascular options reported include retrograde IMA coil embolization via the superior mesenteric artery and Riolan‘s arch to occlude the flow to the inferior caval or right common iliac vein. Although no complications occurred in the reported cases, there may be a risk of pulmonary embolism from aneurysmal thrombus mobilization or wire manipulation.

In the long term, persistent and non-sealed T2ELs can lead to aneurysm growth ≥ 5 mm (9%) with consecutive T1EL (3%) or even T2EL-related secondary re-rupture (6%) [17]. In contrast to these reported rates, Boniakowski et al. did not observe either sac expansion, secondary rupture or aneurysm-related mortality within a mean follow-up of 21 months [22]. The original rupture site usually closes due to the surrounding scarring tissue, but recent morphologic studies have revealed a structural degeneration of the aneurysm wall possibly increasing the risk of T2EL-related aneurysm growth and secondary rupture if they persist over time [36]. Therefore, as in eEVAR, regular CTA and/or CEUS follow-up should be scheduled to detect aneurysm growth promptly. Fortunately, as noted above, most patients (81%) with T2EL after rEVAR will show stable aneurysm sac diameters over time [17].

## 5. Reinterventions and Mortality Related to T2ELs after rEVAR

The reported rate of T2EL-related overall reintervention after rEVAR in two representative studies is about 9–43% within a follow-up time of 21–26 months [17,18] (Table 1).

Due to the above-mentioned risk of persistent hemorrhage, reinterventions for T2ELs after rEVAR may be required more often in the early postoperative period than in the long term: the T2EL-related reintervention rate within 30 postoperative days was already 6–36% [17,18]. However, most reinterventions after rEVAR were not related to T2ELs, but to remaining T1ELs (11%) or ACS (9%) [17]. The role of persisting T2ELs as an underlying cause for the development of an ACS due to persisting hemorrhage and growing hematoma is unclear so far but seems less likely as no association between persistent T2Els after rEVAR and the need for abdominal compartment decompression has been reported.

T2EL-related perioperative mortality after rEVAR is reported to be around 0–6% [17,18]. The reasons for this complication were persistent hemorrhage or secondary rupture due to aneurysm growth, loss of the proximal sealing and consecutive T1EL with possible stent–graft migration.

During follow-up, the overall mortality rate of patients with T2EL after rEVAR is reported to be 59% after 26 months, without a control group of patients without T2ELs in this study [17]. In another study, a comparison of in-hospital (T2EL: n = 1 (6.3%) vs. no T2EL: n = 8 (20%), *p* = 0.23) and estimated overall mortality (*p* = 0.12) between patients with and without T2ELs after rEVAR showed no statistical significance [18].

## 6. Management of Persistent T2ELs after rEVAR

**Figure 1:**
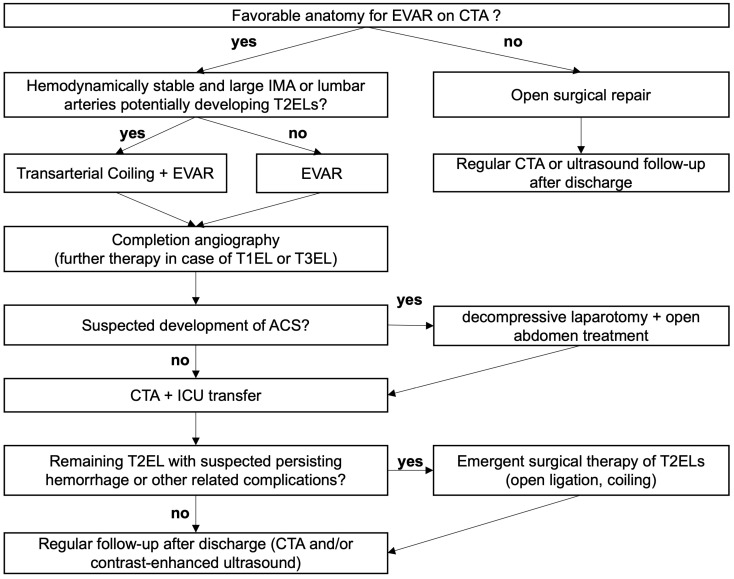
Proposed management of type 2 endoleaks after ruptured endovascular aortic repair.

The management of patients with an rAAA and favorable anatomy for rEVAR depends on their hemodynamic situation, the presence of a patent, large IMA or lumbar arteries, the already intraoperatively suspected development of an ACS as well as on the postoperative persistence of T2ELs and their clinical impact. A flowchart with a proposed management strategy is demonstrated in Figure 1.

The indication for the immediate reintervention of T2ELs after rEVAR is the identification of a relevant T2EL with persistent blood flow and contrast extravasation outside the aneurysm sac with progressive hemodynamic instability of the patient. Some have considered low-flow persistent T2ELs without aneurysm sac growth can be followed without reintervention but require regular imaging control over time. Different concepts for the management of persistent T2ELs after rEVAR with several disadvantages and advantages are discussed in Table 2.

Whenever possible, the authors suggest to prefer a hybrid over a standard operation room with a C-arm angiography system due to the possibility to combine rEVAR with—when necessary—decompressive laparotomy for ACS under optimal conditions and due to the lower contrast agent volume, which might positively influence renal outcomes [37].

### 6.1. Prevention

In the setting of rAAAs, the first aim is to save the patient’s life and to implant the stent–graft as quickly as possible in order to prevent ongoing bleeding from the aortic rupture site. The hemodynamic situation in these patients usually does not allow sometimes time-consuming procedures that are intended to prevent usually benign-characterized T2ELs [38]. However, if the patient is in a hemodynamically stable condition and in the presence of large lumbar arteries and/or an IMA and the surgeon assumes the development of a relevant T2EL, preventive embolization could be a valuable option. Despite that, it should not be considered as a routine procedure.

Very rarely, data on embolization techniques in the acute setting of rupture have been published: Koike et al. presented an intraoperative technique of sac angiography and embolization after successful stent–graft deployment during rEVAR. They introduced a second 0.035-inch guidewire through the contralateral sheath into the aneurysm sac before deploying the contralateral limb and performed a catheter-based angiography of the sac to assess the presence or absence of persistent active bleeding from a T2EL. In the case of active bleeding via T2ELs, N-butyl cyanoacrylate glue was injected into the rupture hole for embolization [20].

Data on other preventive embolization techniques for T2ELs after rEVAR are not available. Therefore, current strategies are based on reports and patient series obtained from studies on T2ELs after eEVAR. Therefore, the prophylactic embolization of lumbar and/or mesenteric arteries, possibly coupled with the embolization of the aneurysm sac itself, before/during the deployment of the abdominal aortic stent–graft is used in some centers based on predefined selection criteria, although the results of these concepts remain controversial [33,39,40]. Guideline recommendations do not exist so far. An overview of the currently available access routes and materials for the embolization of T2ELs is presented in Table 2 and Table 3, respectively [41,42]. Stent–grafts specifically designed for the prevention of T2ELs such as those using polymer-filled endobags have not proven satisfactory and new modifications are under evaluation [43].

### 6.2. Acute Postoperative Phase after rEVAR

If a T2EL is visualized upon the completion of angiography or directly on postoperative CTA after rEVAR, attention should be given not to miss a clinically relevant persistent hemorrhage with blood or contrast extravasation outside the aneurysm sac. This includes close monitoring of the patient‘s hemodynamic situation and hemoglobin levels in short-interval timeframes on the intensive care unit.

If persistent hemorrhage is suspected based on hemodynamic instability, high inotropic support requirements and decreasing hemoglobin levels with the need for serial transfusions, CTA should be emergently performed or repeated. In the case of a corresponding increasing periaortic hematoma or bleeding from lumbar and/or mesenteric arteries completing the diagnosis of a clinically relevant persistent hemorrhage, immediate surgical intervention is indicated. Even without any objective evidence of aneurysm-related hemorrhage on CTA, surgical intervention should be discussed and performed, if persistent hemorrhage is highly suspected even by clinical parameters alone [18]. It has to be borne in mind that the T2EL-related hemorrhage can sometimes be difficult to diagnose due to the large retroperitoneal hematoma and slow blood flow in the lumbar arteries and/or IMA.

In addition, attention should be paid to postoperative clinical and echocardiographic signs of heart failure, probably indicating a previously non-detected aorto-caval fistula maintained by persisting T2ELs.

Reported surgical options for T2ELs after rEVAR include transarterial coil or vascular glue embolization or open surgical ligation of the feeder vessels [17,18]. Some techniques used for T2EL occlusion such as translumbar or transcaval coil embolization have not yet been described in rEVAR. As treatment of T2ELs in the situation of rAAAs takes place in an emergency setting, the most effective option available should be preferred to stop the hemorrhage. Open surgical lumbar artery ligation could be considered as the first choice in patients with concomitant ACS, because decompressive laparotomy is indicated in any case by the current clinical practice guidelines and surgical ligation can then be combined with the evacuation of the hematoma and initiation of temporary abdominal negative wound pressure therapy [16,44]. However, open ligation after rEVAR can be more challenging than after elective EVAR due to the periaortic hematoma and diffuse bleeding in retroperitoneal tissues in the early postoperative phase. In eEVAR, open ligation has a technical success rate of 98% [45], while no data exist for rEVAR. The same challenges apply for laparoscopic lumbar artery or IMA ligation, which has not yet been considered as an effective treatment concept during rEVAR.

### 6.3. Surveillance after Discharge

If no clinically relevant hemorrhage is diagnosed during the early postoperative phase and the patient survives the initial event and hospital stay, close CTA and/or CEUS follow-up after discharge is mandatory. In the absence of specific guideline recommendations for follow-up after rEVAR, established surveillance guidelines such as the detailed algorithm for post-EVAR follow-up from the European Society for Vascular and Endovascular Surgery are recommended [16]. These are mainly focused on whether the aortic anatomy was hostile (i.e., at higher risk for long-term stent–graft-related complications) and the absence or presence of endoleaks on first postoperative CTAs; additional considerations also play a role such as, for instance, whether the stent–graft was delivered within the manufacturer’s instructions for use or not. The role of aneurysm sac regression, which after eEVAR is considered to be correlated with the incidence of reintervention, rupture and mortality, has not yet been investigated for rEVAR [46], but could be identical. To date, there is no evidence for a worse long-term outcome in patients with T2EL after rEVAR that would justify a closer follow-up than usually conducted after eEVAR.

In addition, cardiovascular risk management including blood pressure and lipid control as well as antiplatelet therapy, as recommended by the current clinical practice guidelines after eEVAR, should also be aimed at after rEVAR [16].

Those guidelines, however, do not involve recommendations on anticoagulation therapy. It is known that anticoagulation can influence the outcome in vascular patients such as a higher rate of aortic events in patients with conservatively treated type B aortic dissection [47]. Recent studies on the relation between anticoagulation and T2Els showed a lack of aortic sac reduction and higher rates of T2Els [26,48]. If preexisting, patients‘ indications for anticoagulation should therefore be critically reviewed and anticoagulation therapy should be stopped, if not required.

### 6.4. T2EL-Related Long-Term Complications

In patients with persistent T2ELs after rEVAR and significant aneurysm sac growth during follow-up, the therapeutic options are similar to those in eEVAR patients including transarterial embolization of feeding vessels and endoleak nidus or open surgical ligation of the feeding vessels (Table 3 and Table 4) [17,18].

Although data on the outcomes of these techniques exist for eEVAR patients, not all of them have been described in rEVAR patients. A relevant difference between eEVAR and rEVAR in the long term is the presence of scarred tissue around the prior aortic rupture site and hematoma location, which can challenge open surgical ligation or laparoscopic branch ligation. The latter is an option described for eEVAR, but no data for patients after rEVAR exist. Access to posteriorly located collateral branches may prove especially challenging so—in eEVAR—laparoscopic branch ligation is usually preferred for the treatment of T2ELs originating from the IMA and can be combined with direct sac puncture embolization or intraoperative indocyanine green angiography [49,50,51].

Transabdominal or translumbar direct sac puncture with glue and/or coil embolization is another option for T2EL treatment after eEVAR [52]. A comparative study of direct sac puncture versus transarterial embolization of T2ELs showed similar incidences of aneurysm sac growth, persistent T2EL rates and complications, but a significantly shorter fluoroscopy and total procedure time after direct sac puncture [53].

Once a secondary T1EL has evolved due to the increasing aneurysm sac size and length, or when T2EL sealing proves technically impossible with evidence of continuous sac enlargement, the management options are the same for patients with endograft failure after prior eEVAR. Those include proximal neck fixation with endoanchors, proximal relining with aortic cuffs coupled with chimney stenting, proximal extension with fenestrated or branched devices and open conversion with aortic graft replacement after partial or complete stent–graft explantation [17,54,55,56,57,58,59]. This can also be performed as a prophylactic step if the proximal landing zone is shrinking due to the aneurysm sac increase with impending T1EL [17].

## 7. Conclusions

In summary, the presence of T2ELs after rEVAR should be observed with caution in the early postoperative phase due to potential risks for persistent hemorrhage. In effect, while most of these endoleaks will seal off spontaneously over time, aggressive management may be warranted if persistent T2ELs are suspected to contribute to patients’ poor physiology after rEVAR. Following the exclusion of a type 1 or 3 endoleak, suspicion of a relevant T2EL after rEVAR may be based on unclear blood loss, patients’ hemodynamic instability, retroperitoneal contrast extravasation or the presence of an aorto-caval fistula based on repeated CTA imaging requiring endovascular reintervention or open surgical repair. Preoperative objective data to support preemptive IMA or lumbar artery occlusion for the prevention of T2ELs during rEVAR are not established and remain with the surgeons’ judgement and experience. In the long term, patients with persistent T2ELs after rEVAR should be monitored with at least yearly CTA and/or CEUS, since the initial scarring aneurysm rupture site might be at increased risk of secondary re-rupture. If significant aneurysm sac expansion is detected during follow-up, secondary intervention for persistent T2ELs should be pursued to prevent serious complications. However, several questions remain unanswered and more data for better evidence-based recommendations are needed.

## Figures and Tables

**Table 1:** Results of patients with type 2 endoleaks after endovascular aortic repair of abdominal aortic rupture.

Authors	Year	n	Follow-Up [months]	T2EL	Sealing	T2EL-Related Reinterventions		T2EL-Related Mortality
						30 Days	Overall	
Menges et al. [17]	2023	138	26	35 (25%)	40%	6%	9%	6%
Boniakowski et al. [18]	2016	56	21	16 (29%)	43%	36%	43%	0%

T2EL = type 2 endoleak.

**Table 2:** Discussion of the timing of treatment of type 2 endoleaks after endovascular aortic repair for abdominal aortic rupture.

	Simultaneous Treatment	Delayed Treatment
Advantage	Reliable prevention of T2EL-related complications (persisting hemorrhage, heart failure if aorto-caval fistula is present, sac growth, possibly abdominal compartment syndrome).	Faster procedure time, especially important in hemodynamically stable patients.
	Endovascular techniques are available to identify and treat T2ELs when the aortic stent–graft is already deployed.	As most T2Els resolve over time, only the sourcing arteries can be occluded.
		Higher cost-efficiency.
Disadvantage	Time-consuming and risky in hemodynamically unstable patients.	Risk of T2El-related complications.
	Not always possible (ostial stenosis, unfavorable anatomy).	Patient requires a second procedure.
	Lack of evidence to preoperatively identify T2EL-developing arteries.	
	Risk of spinal cord ischemia.	
Conclusion	Good preventive option that should be limited to hemodynamically stable patients with easily accessible, large arteries.	Preferred option in critical patients but requires close attention to T2EL-related complications such as persisting hemorrhage.

T2EL = type 2 endoleak.

**Table 3:** Access routes for embolization techniques of type 2 endoleaks after endovascular aortic repair [14].

Access Route	Pros	Cons	Materials
Transarterial Cannulation of the target branch vessel (IMA or internal iliac artery); Advancing a microcatheter into feeding vessels and the aneurysm sac; Embolization based on findings.	Embolization of both feeding vessels and endoleak nidus.	Collateral pathways can be long and tortuous and potentially very difficult or impossible to navigate.	Microvascular plugs; Coils; Onyx.
Transcaval Access to the inferior cava vein (ICV) via the internal jugular vein or the common femoral vein; A sheath is pushed up against the wall of the IVC; A dedicated needle is then used to access the aneurysm sac.	Relatively quick to perform.	Potential risks: retroperitoneal hemorrhage, pulmonary embolism and aorto-caval fistula; Only appropriate if the endoleak is abutting on the right side of the aorta; Usually impossible to obtain selective embolization of feeding vessels.	Coils; Onyx.
Perigraft Femoral arterial access; Catheter placement against the distal edge of the endograft in the iliac artery; Wire is forced between the endograft and arterial wall within the aneurysm sac.	When other techniques fail; In recalcitrant endoleaks.	Potentially risks iliac artery dissection; Usually impossible to obtain selective embolization of feeding vessels.	Coils; Onyx.
Translumbar Direct percutaneous puncture; Patient in prone position; Angiogram through the access via a microcatheter; Advancing a microcatheter to the nidus with subsequent embolization.	Relatively quick to perform.	Usually impossible to obtain selective embolization of feeding vessels.	Coils; Onyx.

**Table 4:** Materials for embolization of type 2 endoleaks after endovascular aortic repair [14].

Materials	PROS	CONS
Onyx	Powerful embolic; Flow-directed; Not reliant on coagulation cascade; It can fill the endoleak nidus and the inflow and outflow vessels; Highly radiopaque, with facile monitoring of the injection, potentially decreasing the risk of non-targeted embolization.	Requires experience; Can “glue” catheter in place; Non-target embolization; Long injection times; It is mandatory to use a dimethylsulfoxide-compatible microcatheter.
Coils	Low risk of non-target ischemia; High level of operator control throughout the procedure (especially with detachable coils that can also be retrieved and repositioned); Suitable for embolization of feeding vessels and for filling the endoleak nidus/aneurysm sac; Different lengths/diameters available.	Reliant on coagulation cascade; Can require multiple devices to achieve complete occlusion; Trackability can be an issue especially in highly tortuous vessels.
Microvascular Plugs	Low risk of non-target ischemia; High level of operator control throughout the procedure (be retrieved and repositioned); Does not require large sheaths for delivery; Can embolize vessels up to 9mm.	Occlusion can take time or require coils as well; Trackability can be an issue especially in highly tortuous vessels; Must be placed in a straight segment of vessels; Not suitable for embolization of the endoleak nidus.
